# Proximity Labelling to Quantify Kv7.4 and Dynein Protein Interaction in Freshly Isolated Rat Vascular Smooth Muscle Cells

**DOI:** 10.21769/BioProtoc.4961

**Published:** 2024-03-20

**Authors:** Jennifer van der Horst, Thomas A. Jepps

**Affiliations:** Vascular Biology Group, Department of Biomedical Sciences, University of Copenhagen, Copenhagen, Denmark

**Keywords:** Proximity ligation assay, Vascular smooth muscle cells, Spatial co-localisation, Subcellular localisation, Fluorescence microscopy, Protein interactions

## Abstract

Understanding protein–protein interactions is crucial for unravelling subcellular protein distribution, contributing to our understanding of cellular organisation. Moreover, interaction studies can reveal insights into the mechanisms that cover protein trafficking within cells. Although various techniques such as Förster resonance energy transfer (FRET), co-immunoprecipitation, and fluorescence microscopy are commonly employed to detect protein interactions, their limitations have led to more advanced techniques such as the in situ proximity ligation assay (PLA) for spatial co-localisation analysis. The PLA technique, specifically employed in fixed cells and tissues, utilises species-specific secondary PLA probes linked to DNA oligonucleotides. When proteins are within 40 nm of each other, the DNA oligonucleotides on the probes interact, facilitating circular DNA formation through ligation. Rolling-circle amplification then produces DNA circles linked to the PLA probe. Fluorescently labelled oligonucleotides hybridise to the circles, generating detectable signals for precise co-localisation analysis. We employed PLA to examine the co-localisation of dynein with the Kv7.4 channel protein in isolated vascular smooth muscle cells from rat mesenteric arteries. This method enabled us to investigate whether Kv7.4 channels interact with dynein, thereby providing evidence of their retrograde transport by the microtubule network. Our findings illustrate that PLA is a valuable tool for studying potential novel protein interactions with dynein, and the quantifiable approach offers insights into whether these interactions are changed in disease.

## Background

Studying protein–protein interactions is crucial for unravelling the subcellular distribution of proteins, contributing to our understanding of cellular organisation. Besides revealing the spatial arrangement of proteins, interaction or co-localisation studies provide insights into the functional relationships between proteins and cellular processes. These studies play a role in clarifying signalling pathways, as the interaction partners of certain proteins often indicate their involvement in specific signalling cascades. Additionally, co-localisation studies can contribute to the elucidation of mechanisms governing the trafficking of proteins within cells. For example, identifying whether a certain protein interacts with the microtubule network or specific motor proteins, such as kinesin or dynein that are responsible for cargo movement along the microtubule network in opposite directions, provides insights into trafficking dynamics, including the direction of trafficking.

Various techniques exist for detecting protein–protein interactions in different cellular contexts, including live and fixed cells, tissues, and protein lysates. These techniques typically involve the use of antibodies or direct fluorescent protein fusions. Förster resonance energy transfer (FRET) is a well-established technique for measuring co-localisation in live cells by expressing two proteins of interest, each fused to different fluorophores (donor and acceptor) [1,2]. Proximity of the two proteins induces energy transfer between the fluorophores, which can be measured. However, FRET has limitations, requiring cultured cells and molecular cloning for the fluorophore fusion to proteins. Co-immunoprecipitation, another method for studying protein–protein interactions, involves using antibodies to pull out targeted proteins from cell or tissue lysates, along with some of the direct interacting partners [3]. Analysis using western blotting or mass spectrometry can identify the binding partners of the targeted protein. However, the success of co-immunoprecipitation can be affected by the choice of lysis buffer since this can easily disrupt several protein–protein interactions. Thus, this technique is not optimal for low-affinity interactions. Additionally, co-immunoprecipitation lacks detailed information about the spatial co-localisation of proteins within a cell. Fluorescence microscopy is a widely used technique, which involves labelling proteins with specific antibodies and fluorescent labels, with co-localisation assessed by evaluating the spatial overlap of two fluorescent signals. Although this technique does not detect direct physical protein–protein interactions, advanced light microscopy techniques, such as stimulated emission depletion or structured illumination microscopy, enhance the resolution, providing a more precise determination of protein co-distribution.

The in situ proximity ligation assay (PLA) enables accurate, spatial detection of protein–protein interactions in fixed cells and tissues [4]. This technique involves using two primary antibodies for the targeted proteins, raised in different species. The species-specific secondary PLA probes, each linked to a unique DNA oligonucleotide, bind to their respective primary antibodies. When the targeted proteins are within 40 nm of each other, the DNA oligonucleotides on the PLA probes interact and serve as a template for the hybridising connector oligonucleotide, resulting in the formation of circular DNA through enzymatic ligation. This circular DNA template undergoes rolling circle amplification, producing DNA circles linked to the PLA probe. Fluorescently labelled oligonucleotides are added, which hybridise to the complementary circle DNA. The resulting fluorescent spots can be visualised using fluorescent microscopy. These detected signals can be quantified and attributed to specific subcellular locations, to determine the co-localisation pattern within cells. Overall, PLA offers a precise way to study the spatial relationships between proteins in cells. Moreover, PLA enables the application of pharmacological treatments to isolated vascular smooth muscle cells prior to fixation, allowing exploration of treatment impacts on the co-localisation of specific proteins. In our study, we employed PLA to visualise the interaction of the motor protein dynein with voltage-gated potassium Kv7.4 channels in freshly isolated vascular smooth muscle cells from rat mesenteric arteries [5]. Our goal was to explore dynein's impact on the microtubule-dependent trafficking of Kv7.4 channels. The Kv7.4 and Kv7.5 channels are important regulators of the resting membrane potential in smooth muscle cells, particularly from arteries [6]. Additionally, these channels contribute to the β-adrenoceptor-mediated vasodilatation in vascular smooth muscle cells [7] and other cAMP and cGMP-derived relaxations [8,9]. In arteries from hypertensive rodents, the Kv7.4 channel is downregulated and function is attenuated [10], which contributes to the reduced β-adrenoceptor-mediated vasodilatation observed in these arteries [11]. Our previous studies indicate that dynein motor proteins facilitate the trafficking of Kv7.4 channels along the microtubule network, away from the plasma membrane, in rat vascular smooth muscle cells [5,12]. Furthermore, manipulating this trafficking system by inhibiting the dynein-mediated trafficking of Kv7.4 channels enhances Kv7.4 membrane abundance, restoring the β-adrenoceptor-mediated vasodilatation in arteries from hypertensive rats [13].

## Materials and reagents

**Biological materials**

1. Rat (Janvier Labs, France)

This protocol has been employed on male and female rats, including strains of Wistar Kyoto, Wistar Hannover, and Spontaneously Hypertensive rats, aged between 10 and 18 weeks. It is anticipated that this protocol is applicable to rats of other strains and ages.

**Reagents**

1. Milli-Q water
2. Sodium chloride (NaCl) (Sigma-Aldrich, catalog number: S9888), stored at room temperature
3. Sodium gluconate (C_6_H_11_NaO_7_) (Sigma-Aldrich, catalog number: S2054), stored at room temperature
4. Potassium chloride (KCl) (Sigma-Aldrich, catalog number: 12636), stored at room temperature
5. Magnesium chloride hexahydrate (MgCl_2_·6H_2_O) (Sigma-Aldrich, catalog number: M2670), stored at room temperature
6. Calcium chloride (CaCl_2_) (Sigma-Aldrich, catalog number: C1016), stored at room temperature
7. Glucose (C_6_H_12_O_6_) (Sigma-Aldrich, catalog number: G7021), stored at room temperature
8. HEPES (Sigma-Aldrich, catalog number: H3375), stored at room temperature
9. Bovine serum albumin (BSA) (Sigma-Aldrich, catalog number: A9647), stored at 4 °C
10. 1,4-dithiothreitol (DTT) (Sigma-Aldrich, catalog number: DTT-RO), stored at 4 °C
11. Papain from papaya latex (Sigma-Aldrich, catalog number: P4762), stored at -20 °C
12. Collagenase from *Clostridium histolyticum* Type F (Collagenase F) (Sigma-Aldrich, catalog number: C7926), stored at -20 °C
13. Collagenase from *Clostridium histolyticum* Type H (Collagenase H) (Sigma-Aldrich, catalog number: C8051), stored at -20 °C
14. Triton-X (Sigma-Aldrich, catalog number: T8787), stored at room temperature
15. 4% paraformaldehyde (PFA) (Sigma-Aldrich, catalog number: HT501128), stored at 4 °C
16. Phosphate buffered saline (PBS) (Gibco, catalog number: 10010023, or any other PBS without magnesium and calcium), stored at room temperature
17. Rabbit anti-Kv7.4 antibody (Abcam, catalog number: ab65797), stored at -20 °C
18. Mouse anti-Dynein antibody (Abcam, catalog number: ab23905), stored at -20 °C
19. Duolink in situ PLA probe anti-mouse PLUS (Sigma-Aldrich, catalog number: DUO92001-100RXN), stored at 4 °C
20. Duolink in situ PLA probe anti-rabbit MINUS (Sigma-Aldrich, catalog number: DUO92005-100RXN), stored at 4 °C
21. Duolink in situ detection reagents red, with 5× ligation buffer and5× amplification buffer (Sigma-Aldrich, catalog number: DUO92008-100RXN), stored at -20 °C
22. Duolink in situ wash buffer A and wash buffer B (Sigma-Aldrich, catalog number: DUO82049-4L), stored at room temperature or at 4 °C once dissolved (see Recipes)
23. Anti-dynein (Mouse; 1:500: ab23905; Abcam) and anti-Kv7.4 (Rabbit; 1:200; ab65797; Abcam)

**Solutions**

1. Calcium chloride (1 M) (see Recipes)
2. Calcium chloride (100 mM) (see Recipes)
3. Magnesium chloride (1 M) (see Recipes)
4. HEPES-Krebs solution (see Recipes)
5. Smooth muscle cell dissection solution (SMDS) (see Recipes)
6. Stock solutions (see Recipes)
7. Tube 1 (see Recipes)
8. Tube 2 (see Recipes)

**Recipes**

Prepare all solutions in volumetric flasks up to the specified volume. Using volumetric flasks will contribute to precise measurements and reliable results in your experiments.

1. **Calcium chloride (1 M)**
  **Table:** 

Reagent	Final concentration	Quantity or Volume
Calcium chloride	1 M	11.098 g
  Dissolve the calcium chloride in Milli-Q water, add the volume up to exactly 100 mL, and store at 4 °C.
2. **Calcium chloride (100 mM)**
  Dilute 100 μL of calcium chloride 1 M in 900 μL of Milli-Q water and store in a 1.5 mL tube at room temperature.
3. **Magnesium chloride (1 M)**
  **Table:** 

Reagent	Final concentration	Quantity or Volume
Magnesium chloride hexahydrate	1 M	20.33 g
  Dissolve the magnesium chloride hexahydrate in Milli-Q water, add the volume up to exactly 100 mL, and store at 4 °C.
4. **HEPES Krebs solution**
  **Table:** 

Reagent	Final concentration	Quantity or Volume
Sodium chloride	134 mM	1.593 g
Potassium chloride	6 mM	89.46 mg
Glucose	7 mM	252.21 mg
HEPES	10 mM	476.6 mg
Calcium chloride (1 M)	2 mM	400 μL
Magnesium chloride (1 M)	1 mM	200 μL
  Dissolve the reagents in Milli-Q water, adjust the pH to 7.4, and add the volume up to exactly 200 mL. The solution should be freshly prepared on the day of experiment.
5. **SMDS solution**
  **Table:** 

Reagent	Final concentration	Quantity or Volume
Sodium chloride	60 mM	1.75 g
Sodium gluconate	80 mM	8.73 g
Potassium chloride	5 mM	186.38 mg
Magnesium chloride hexahydrate	2 mM	203.3 mg
Glucose	10 mM	900.75 mg
HEPES	10 mM	1.19 g
  Dissolve the reagents in Milli-Q water, adjust the pH to 7.4, and add up to exactly 500 mL. After preparation, distribute the solution into 50 mL tubes and store them at -20 °C. Thaw one 50 mL SMDS solution on the day of the experiment before use.
6. **Wash buffer A and wash buffer B**
  Prepare PLA wash buffer A and B ahead by dissolving the PLA buffer sachets each in 1 L of Milli-Q. Filter sterilise both buffers using a syringe and a 0.2 μm pore filter and store at 4 °C.
7. **Stock solutions**
  **Table:** 

Reagent	Concentration	Minimum volume needed
BSA	10 mg/mL	200 μL
DTT	10 mg/mL	150 μL
Papain	5 mg/mL	100 μL
Collagenase F	10 mg/mL	70 μL
Collagenase H	5 mg/mL	40 μL
8. **Tube 1**
  **Table:** 

Stock solution	Final concentration	Quantity or Volume
BSA (10 mg/mL)	1 mg/mL	100 μL
DTT (10 mg/mL)	1.5 mg/mL	150 μL
Papain (5 mg/mL)	0.5 mg/mL	100 μL
SMDS	n/a	650 μL
Total	n/a	1,000 μL
9. **Tube 2**
  **Table:** 

Stock solution	Final concentration	Quantity or Volume
BSA (10 mg/mL)	1 mg/mL	100 μL
Collagenase F (10 mg/mL)	0.7 mg/mL	70 μL
Collagenase H (5 mg/mL)	0.2 mg/mL	40 μL
*Calcium Chloride (100 mM)	100 µM	1 μL
SMDS	n/a	790 μL
Total	n/a	1000 μL
  *A stock solution of calcium chloride can be pre-made and stored at room temperature for repeated use.

**Laboratory supplies**

1. Surgical scissors (Fine Science Tools, Mayo-Stille Scissors, catalog number: 14101-14)
2. Dissection forceps (Fine Science Tools, Fine Forceps, catalog number: 11252-00)
3. Dissection scissors (Fine Science Tools, Spring scissors, catalog number: 15020-15)
4. Dissection dish (Silicone coated)
5. Sewing pins
6. 1.5 mL microcentrifuge tube (Eppendorf, catalog number: 0030120086)
7. 50 mL Falcon tube (Falcon)
8. 100/200 mL glass beaker
9. 50 mL syringe (BD Plastipak)
10. 0.2 μm pore syringe filter (Sigma-Aldrich, catalog number: WHA9914)
11. Disposable Pasteur pipette (Thermo Fisher, catalog number: 202-1SPK)
12. 24-well cell culture plate (Thermo Fisher, catalog number: 144530)
13. Styrofoam ice box
14. Fire-polished glass pipette (custom-made, see Figure 1)
  **Figure 1.**
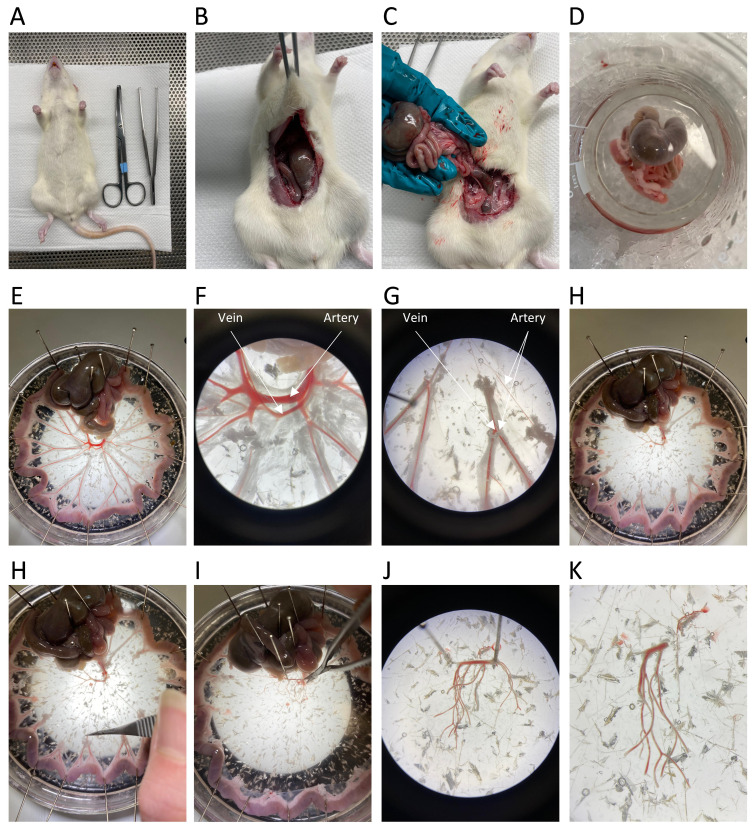
Fire-polishing of the glass Pasteur pipette. A. Remove and discard the long end of the pipette. B. Rotate the end of the pipette in a Bunsen flame until its edges are polished and the opening is smoothed and narrowed. Comparison of the opening before (left) and after (right) fire polishing.
15. Humidified chamber (custom-made)
16. Cover glass 12-mm circles (VWR, catalog number: 76355-906)
17. Microscope slides (VWR, catalog number: 16004-422)
18. Clear nail polish (any)

## Equipment

1. Pipettes (ranging from 1 to 1,000 μL)
2. Heat block (37 °C)
3. Bunsen burner
4. Plate rocker at room temperature
5. 37 °C, 5% CO_2_ cell culture incubator
6. Dissecting microscope (Olympus SZX7 Stereomicroscope)
7. Cell microscope (Leitz Labovert inverted light microscope, 10× objective)
8. ZEIS LSM780/LSM900 microscope or any other laser scanning confocal microscope

## Software

1. ImageJ (version 1.53k, July 2021)
2. GraphPad PRISM (version 9, October 2020)

## Procedure

**Part I: Isolation of rat vascular smooth muscle cells**

- **Preparation**
  1. Take a 50 mL Falcon tube with SMDS solution out of the -20 °C freezer and thaw on ice.
  2. Prepare the HEPES-Krebs solution (see Recipes)
  3. Take the enzymes out of the -20 °C freezer and thaw at room temperature.
- **Isolation of the rat mesenteric arteries**
  1. Sacrifice the rat (in accordance with annex IV of the EU Directive 2010/63EU on the protection of animals used for scientific purposes).
    - Make the rat unconscious by a single percussive blow to the head. Immediately after the onset of unconsciousness, perform cervical dislocation to complete the killing.
  2. Perform a laparotomy using surgical scissors and elevate the intestines out of the abdominal cavity (Figure 2A–2D).
    **Figure 2.**
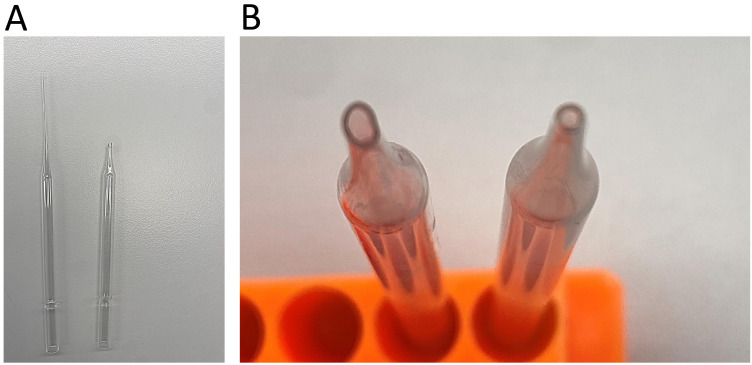
Dissection process of rat mesenteric arteries. A. The male Wistar rat is euthanised via cervical dislocation. B–D. A laparotomy is performed, and the rat intestines are isolated. E. The intestines are pinned onto a dissection dish using sewing pins to expose the mesenteric vessels. F–H. The main mesenteric artery and vein, surrounded by adipose tissue, are visualised. I–K. The surrounding tissue is removed, and 3–4 branches of the mesenteric arteries are isolated for the extraction of arterial smooth muscle cells.
  3. Cut the intestines just above the rectum and below the stomach and place the intestines in a beaker filled with HEPES-Krebs placed on ice (Figure 2A –2D).
  4. Place the intestines into a dissection dish filled with cold HEPES-Krebs buffer and pin the jejunum and ileum along the side of the dissection dish forming a circle, allowing visualisation of the mesenteric vessels, with the main arterial branch in the centre (Figure 2E).
  5. Place the dish under a 10× dissecting microscope for visualisation of the arteries and veins (Figure 2F–2G).
    - Arteries can be differentiated from veins by their thicker smooth muscle layer, which makes them more rigid compared to veins. Furthermore, the thicker wall creates the appearance of a reduced blood volume compared to veins.
  6. Remove all tissue surrounding the arteries (Figure 2G–2H).
    - Begin by locating the larger veins and carefully tear them using two forceps; then, remove them all the way down to the lower-order branches.
    - Cut through the mesentery connecting the arterial branches and remove all adipose tissue surrounding the arteries.
  7. Remove the mesenteric arteries by first cutting at the lower-order branches and then free the mesenteric arteries by cutting the central branch (Figure 2I –2K).
  8. Take a small section of the main artery, including 3–4 branches of lower-order mesenteric arteries and place it in a 1.5 mL tube filled with SMDS solution on ice.
  9. Remove the remaining intestines from the dissection dish for disposal.
- **Isolation of the mesenteric arterial smooth muscle cells**
  1. Have a fire-polished glass Pasteur pipette ready. To make a fire-polished pipette, break off the long end of the pipette and throw it out. Expose the pipette with its broken end to a Bunsen flame, constantly rotating it. Continue with this process until the edges are polished and the opening is smoothed and narrowed to an inner diameter of roughly 0.7–1 mm (Figure 1). Wash the pipette with ethanol after each experiment so it can be used for subsequent future experiments.
  2. The following solutions used for smooth muscle cell isolation have been adapted from Zhong et al. [14] and Chadha et al. [11]. Prepare the following stock solutions (Recipes 7–9) by dissolving them in SMDS solution (Recipe 5) in 1.5 mL tubes and keep them at room temperature.
    - These stock solutions have to be prepared fresh on the day of the experiment and cannot be stored for future experiments.
  3. Place the tube with the mesenteric artery branch in SMDS solution in a 37 °C heat block for 10 min to equilibrate the arteries at 37 °C.
  4. In the meantime, prepare Tube 1 (Recipe 8) by combining the stock solutions (Recipe 7) and SMDS solution in a 1.5 mL tube.
  5. Transfer the mesenteric arteries to Tube 1. Gently invert the tube 4–5 times to ensure proper mixing of the arteries with the solution and incubate at 37 °C for 10 min.
  6. In the meantime, prepare Tube 2 (Recipe 9) by combining the stock solutions (Recipe 7) and SMDS solution in a 1.5 mL tube.
  7. Take the arteries from the heat block and wash five times in ice-cold SMDS solution using a glass Pasteur pipette with a rubber bulb.
  8. Transfer the mesenteric arteries to Tube 2. Gently invert the tube 4–5 times to mix the arteries with the solution, and incubate at 37 °C for 10 min.
    - The arterial branch may appear slightly blurry or fluffy during this step; this means that the enzymatic digestion is effectively isolating the cells.
  9. Gently wash five times in ice-cold SMDS solution using a glass Pasteur pipette with a rubber cap. During the last wash, replace the solution with 500 μL of SMDS.
  10. Liberate the myocytes by gently triturating the arterial branch using the custom-made fire-polished glass pipette.
    - Perform approximately 30 up-and-down triturations.
  11. Store the supernatant containing liberated myocytes in a separate tube on ice and add 500 μL of fresh SMDS to the arterial branch in the tube. Place a droplet of the supernatant on a glass slide and assess the cell density using a 10× microscope.
  12. Repeat the trituration process and save the supernatant in separate tubes on ice, until no more cells are released within the supernatant.
    - This process can take approximately 1–5 rounds of trituration.
*Note: Consider shortening the artery incubation time (1–3 min shorter) with enzymes in tube 1 and tube 2, if the enzymes are newly opened, to prevent over-digestion of the arteries and enhance myocyte liberation.*
  13. Combine the supernatants that have liberated myocytes and adjust the concentration of cells by adding more SMDS to achieve the desired cell density. By placing 50 µL of the cells on a glass coverslip, it is possible to check the cell density under a light microscope. There is no defined cell density necessary for undertaking this protocol, but smooth muscle cells should be visible under a light microscope’s 20× objective, and the cells should not be crowded and touching one another.
  14. Add 1 μL of 100 mM calcium chloride for every 1 mL of cell suspension (resulting in a final concentration of 100 μM calcium chloride). Calcium is required for the cells to adhere to the coverslips.
*Note: Consider increasing the calcium chloride concentration by 10%–20% if there is significant loss of cells from the coverslip after fixing the cells.*
  15. Add 100 μL of cell suspension on a 12 mm coverslip in a 24-well plate and keep at room temperature for 30 min to let the cells adhere to the coverslips (Figure 3A ).
    - After 30 min, the cells can be incubated with a pharmacological agent. To do this, dissolve the compound at the desired concentration in SMDS solution supplemented with 100 μM calcium chloride, replace the solution on the coverslip with 100 μL of compound solution, and incubate at 37 °C.
    **Figure 3.**
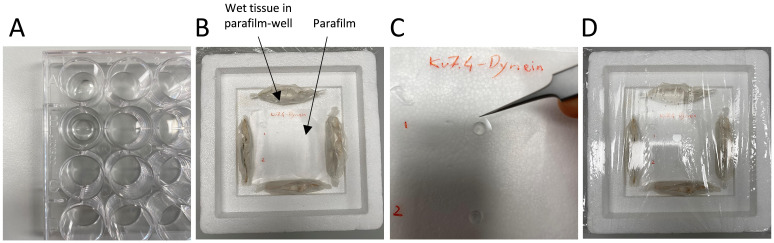
Adhesion and primary antibody incubation for isolated vascular smooth muscle cells. A. Place 100 µL of cell suspension onto each coverslip in the well. B. Create a humidified chamber using a Styrofoam icebox and parafilm. C. Incubate the cells with the primary antibody by applying a drop of the diluted antibody on the parafilm and placing the coverslip with adhered cells on top. D. Seal the humidified chamber with cling film for incubation at 4 °C.
  16. Fix the cells by gently aspirating the SMDS solution from the coverslip and apply 400 μL of 4% PFA to each well. Place the 24-wells on the rocker for 15 min at room temperature.
  17. Wash three times with PBS, 400 μL per well.
  18. Use the coverslips immediately for PLA experiment or store coverslips in the 24-wells plate (with each well containing 1 mL of PBS and sealed with parafilm) at 4 °C for up to four weeks until used for the PLA experiment. The duration of storage depends on the specific cell type.

**Part II: PLA**

- **Preparation**
  1. Allow the PLA wash buffers A and B to reach room temperature prior to use, as cold wash buffers can lead to nonspecific background signal.
- **Permeabilization, blocking, and primary antibody incubation**
  1. Prepare a sufficient volume of 0.1% Triton-X in PBS to add 400 μL of the solution to each coverslip within the well.
  2. Add 400 μL of 0.1% Triton-X to each well, place on the rocker, and incubate for 5 min to permeabilise the cells.
  3. Wash two times for 5 min each in PBS.
  4. Remove the PBS and apply three drops of the blocking buffer (provided in the PLA probe kit) onto the cells on the coverslip in each well. Incubate at 37 °C for 30 min.
  5. Prepare the primary antibody mix. It is very important that the two primary antibodies targeting the protein of interest are raised in different species.
    - In our experiment, to look at Kv7.4 and dynein co-localisation, we prepared a primary antibody mixture consisting of anti-dynein and anti-Kv7.4 antibodies.
  6. Dilute the primary antibodies in the antibody diluent provided in the PLA probe kit, following the specific requirements of each antibody.
    - Prepare enough to use 40 μL of the primary antibody mix for each coverslip.
  7. Create a humidified chamber (Figure 3B).
    - Use the lid of a Styrofoam ice box; the lid must have a recessed area in the middle.
    - Place a piece of parafilm in the centre of the lid, where the coverslips will be placed for incubation.
    - Create four wells using parafilm, each well containing a wet tissue inside (see Figure 3B). Place the four wet tissues in parafilm wells around the flat piece of parafilm in the middle of the Styrofoam lid. This will help maintain moisture and prevent the coverslips from drying out during the overnight incubation with the primary antibodies.
  8. Add a 40 μL drop of primary antibody mix to the parafilm.
  9. Using forceps, lift the coverslip from the blocking solution, removing it from the well. Gently tap the side of the coverslip on a paper tissue to remove excess blocking buffer and place it over the antibody drop with the side containing cells making contact with the drop and facing downward (Figure 3C).
  10. Cover the Styrofoam lid with cling film and leave it overnight at 4 °C (Figure 3D).
*Note: This primary antibody concentration and incubation time may require optimisation.*
- **Probe incubation**
*Note: Each coverslip represents one reaction, and 40 µL of solution is used per reaction. The calculations provided in the following steps are for a single reaction.*
  1. The following day, take the PLA PLUS and MINUS probe out of the fridge and vortex.
  2. Prepare the PLA probe mix by diluting the PLA PLUS and MINUS probes 1:5 in the antibody diluent provided in the kit. Make sufficient PLA probe solution to use 40 μL per coverslip.
    - For one coverslip (40 μL): dilute 8 μL of PLUS and 8 μL of MINUS in 24 μL of antibody diluent.
  3. Place the coverslips incubated with the primary antibodies back in the 24-well plate and wash two times for 5 min each in PLA wash buffer A at room temperature on a rocker.
  4. Place a new piece of parafilm in the handcrafted humidified chamber and add 40 μL drops of PLA probe mix for every coverslip.
  5. Add the coverslips on top of the droplets with the side containing cells facing downward.
  6. Place the humidified chamber in a 37 °C incubator for 1 h.
- **Ligation**
  1. Place the coverslips back in the 24-well plate and wash two times for 5 min each in PLA wash buffer A at room temperature on a rocker.
  2. Prepare the PLA ligation mix by diluting the 5× ligation buffer with Milli-Q water (1:5 dilution) and add the ligase (1:40 dilution) to the mix.
    - For one coverslip (40 μL): dilute 7.8 μL of 5× ligation buffer in 31.2 μL of Milli-Q water and 1 μL of ligase.
    - Wait to add the ligase until immediately prior to incubation.
  3. Place a new piece of parafilm in the handcrafted humidified chamber and add 40 μL droplets of PLA ligation mix for every coverslip.
  4. Add the coverslips on top of the droplets with the side containing cells facing downward.
  5. Place the humidified chamber in a 37 °C incubator for 30 min.
- **Amplification**
*Note: The fluorophore in the Amplification Red detection kit has an excitation/emission wavelength of 594 nm/624 nm, respectively, and can be detected using the Texas Red filter (see Part III.). Alternative detection fluorophores are also available, such as Amplification Green, Orange, and Far Red.*
  1. Place the coverslips back in the 24-well plate and wash twice for 2 min each time in PLA wash buffer A at room temperature on a rocker.
  2. Prepare the PLA amplification mix by diluting the 5× amplification buffer with Milli-Q water (1:5 dilution) and add the polymerase (1:80 dilution) to the mix.
    - For one coverslip (40 μL): dilute 7.9 μL of 5× amplification buffer in 31.6 μL of Milli-Q water and 0.5 μL of polymerase.
    - Wait to add the polymerase until immediately prior to incubation.
  3. Place a new piece of parafilm in the handcrafted humidified chamber and add 40 μL droplets of PLA amplification mix for every coverslip.
  4. Add the coverslips on top of the droplets with the side containing cells facing downward.
  5. Place the humidified chamber in a 37 °C incubator for 100 min, protected from light.
- **Mounting**
  1. Place the coverslips back in the 24-well plate and wash twice for 10 min each time in PLA wash buffer B at room temperature on a rocker.
    - Protect the samples from light by placing aluminium foil over the 24-well plate.
  2. Wash for 1 min in 0.01× PLA wash buffer B.
  3. Mount coverslips on microscope slides using 3 μL of mounting media containing DAPI for each coverslip.
  4. Let the coverslips dry at room temperature protected from light for 10 min.
  5. Use clear nail polish to seal the edges of the coverslip to the slide. Avoid getting air bubbles caught under the coverslip.
  6. Store the slides in the dark at 4 °C for up to four days or at -20 °C for six months.

**Part III: Imaging and analysis**

- **Imaging**
  1. Visualise fluorescent signal by a confocal microscopy (Figure 4).
    **Figure 4.**
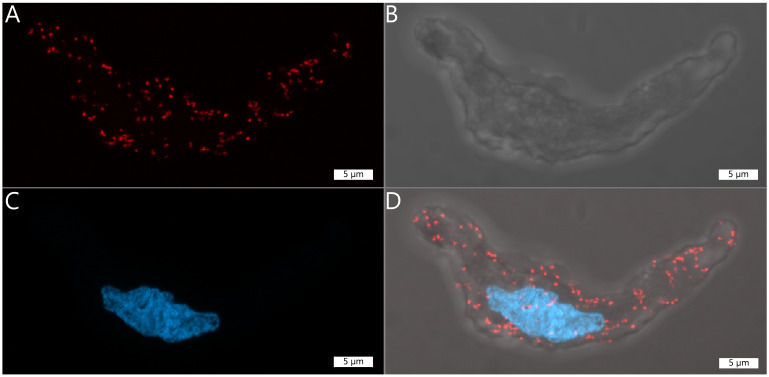
Representative images of a single vascular smooth muscle cell displaying a number of proximity ligation assay (PLA) dots. Each red dot represents a site where the two primary antibodies have bound to their respective protein within 40 nm of each other. A. Texas Red channel (excitation wavelength 592), in which the PLA dots are visible. B. Brightfield image of the vascular smooth muscle cell. C. DAPI staining of the nucleus (excitation wavelength 353). D. Merged image of A, B, and C. Scale = 5 µm
  2. Use appropriate microscope settings.
    - In our specific setup, we capture images using a 63× oil immersion objective on either a ZEIS LSM780 or LSM900 laser scanning confocal microscope.
    - Choose a DAPI filter for nucleus detection and a Texas red filter for PLA dot detection. To visualise cell contours and contrast, include a brightfield imaging channel in your microscopy setup (Figure 4).
    - Ensure consistent laser settings are applied for each experiment.
  3. Capture full z-stack images of individual cells. Multiple cells can be captured per coverslip.
  4. For quantitative comparisons, PLA signals of at least 30 cells are captured from at least three biological replicates.
    - Include a negative control sample as part of the experiment. A suitable negative control may involve the use of two protein-targeting antibodies that are known to have no co-localisation and, therefore, should not produce a signal. Alternatively, consider using protein knockdown cells for additional control measures. Another appropriate control, as implemented in our study, involves the use of only one of the primary antibodies.
- **Data analysis**
*Note: Analyse the number of fluorescent PLA dots within each cell. Depending on the desired approach, PLA dots can be quantified on a single mid-cell z-plane or conducted on a maximum intensity projection encompassing the entire cell.*
  1. Open the image file in ImageJ. Make sure to open the images for PLA dots, DAPI, and brightfield in separate windows instead of using the merged channels file. You can achieve this by selecting the *Split channel* option (Figure 5A ).
    **Figure 5.**
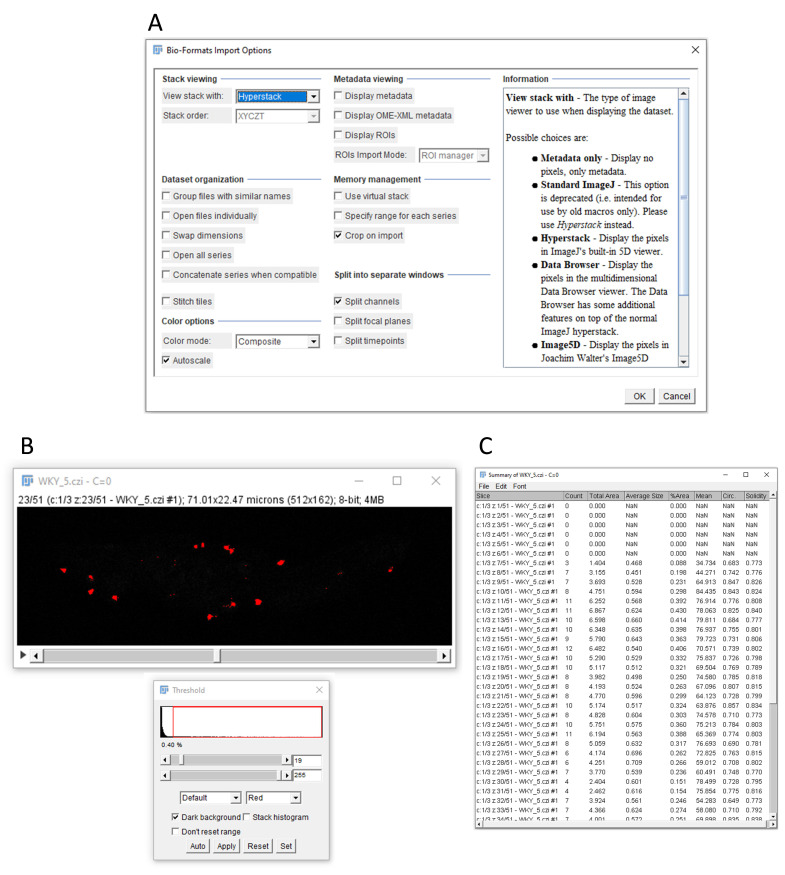
Data analysis in ImageJ. A. Specific settings for opening a microscopy image in ImageJ. B. Example of threshold parameters for visualizing clear dots while minimizing background. C. Example of the data output, showing the number of dots per z-stack, with each row corresponding to a different stack.
  2. Three individual windows, representing the PLA dots, DAPI, and brightfield will now open. Close the DAPI and brightfield windows so that only the PLA dots window remains visible.
  3. In the ImageJ browser, select *Image* > *Adjust* > *Threshold*.
  4. Adjust the threshold parameters by clicking and dragging the upper bar from the left to the right until the dots become clearly visible and the background is removed. Ensure that the lower bar is set all the way to the right (Figure 5B ).
  5. To count the number of dots, select in the ImageJ browser *Analyze* > *Analyze particles*.
  6. Adjust the size parameters for particle analysis, adjusting both the minimum and maximum size.
    - For our experiments, we typically select a particle size range from 0.3 to 3 micron^2. Particles outside of this range will be excluded from the count. Adjust these parameters as per specific requirements.
  7. Select the *Summarize* option and click *OK.*
    - To mark the counted particles in your PLA image window, select the *Add to manager* option before clicking *OK*.
  8. ImageJ will now give an option to process all images. By selecting *Yes*, ImageJ will count the PLA dots for each stack and generate an output data file for the number of dots per stack (Figure 5C). In case the number of PLA dots for one specific stack should be counted, select *No*, and ImageJ will count the dots for the stack you have currently selected in your PLA image window.
  9. To compare the average number of PLA dots for each cell under various conditions (such as control or pharmacologically treated cells), add the dot counts for each biological replicate into GraphPad PRISM. Analyse the data set using a nested t-test to test whether pharmacological treatment has an impact on the number of interactions between two proteins.

## Validation of protocol

This protocol or parts of it has been used and validated in the following research article(s):

- van der Horst et al. (2021). Dynein regulates Kv7.4 channel trafficking from the cell membrane. J. Gen. Physiol. (Figures 3F, 6C, 7B&C and 8A).
